# Improving the Understanding of Test Results by Substituting (Not Adding) Goal Ranges: Web-Based Between-Subjects Experiment

**DOI:** 10.2196/11027

**Published:** 2018-10-19

**Authors:** Aaron M Scherer, Holly O Witteman, Jacob Solomon, Nicole L Exe, Angela Fagerlin, Brian J Zikmund-Fisher

**Affiliations:** 1 Department of Internal Medicine University of Iowa Iowa City, IA United States; 2 Department of Family and Emergency Medicine Université Laval Quebec City, QC Canada; 3 Office of Education and Professional Development Faculty of Medicine Université Laval Quebec City, QC Canada; 4 Population Health and Optimal Health Practices Research Unit Research Centre of the CHU de Québec-Université Laval Quebec City, QC Canada; 5 Center for Bioethics and Social Sciences in Medicine University of Michigan Ann Arbor, MI United States; 6 Department of Population Health Sciences University of Utah Salt Lake City, UT United States; 7 Salt Lake City VA Informatics Decision-Enhancement and Analytic Sciences (IDEAS) Center for Innovation Salt Lake City, UT United States; 8 Department of Health Behavior and Health Education University of Michigan Ann Arbor, MI United States; 9 Department of Internal Medicine University of Michigan Ann Arbor, MI United States

**Keywords:** decision making, education of patients, electronic health record, computer graphics, clinical laboratory information systems

## Abstract

**Background:**

Most displays of laboratory test results include a standard reference range. For some patients (eg, those with chronic conditions), however, getting a result within the standard range may be unachievable, inappropriate, or even harmful.

**Objective:**

The objective of our study was to test the impact of including clinically appropriate goal ranges outside the standard range in the visual displays of laboratory test results.

**Methods:**

Participants (N=6776) from a demographically diverse Web-based panel viewed hypothetical glycated hemoglobin (HbA_1c_) test results (HbA_1c_ either 6.2% or 8.2%) as part of a type 2 diabetes management scenario. Test result visual displays included either a standard range (4.5%-5.7%) only, a goal range (6.5%-7.5%) added to the standard range, or the goal range only. The results were displayed in 1 of the following 3 display formats: (1) a table; (2) a simple, two-colored number line (simple line); or (3) a number line with diagnostic categories indicated via colored blocks (block line). Primary outcome measures were comprehension of and negative reactions to test results.

**Results:**

While goal range information did not influence the understanding of HbA_1c_=8.2% results, the goal range only display produced higher levels of comprehension and decreased negative reactions to HbA_1c_=6.2% test results compared with the no goal range and goal range added conditions. Goal range information was less helpful in the block line condition versus the other formats.

**Conclusions:**

Replacing the standard range with a clinically appropriate goal range could help patients better understand how their test results relate to their personal targets.

## Introduction

In an effort to facilitate greater patient involvement in the management of their health, hospitals and health care systems have increasingly provided patients with access to their electronic health records (EHRs) [1]. However, simply providing health information, such as laboratory test results, is often insufficient in enabling patients to understand, much less utilize, this information. Test results are commonly presented in a table format, which leaves a sizable minority of people having difficulty with the seemingly simple task of identifying whether their test result falls within the standard range [2]. Furthermore, even when people can correctly identify the location of their test result in reference to the standard range, they tend to view the risk associated with their test value in a dichotomous fashion—with results within the standard range being viewed as “good” and results outside of the standard range as “bad”—without sensitivity to the fact that risk usually changes in a linear or exponential fashion [2].

Individuals who manage chronic conditions face an additional barrier to understanding and effectively using their test results: inappropriate reference ranges. The standard range commonly presented as part of test result communications represents the distribution of values commonly observed in a healthy population [3-5]. In some chronic disease situations, however, the practical target range that the patient and clinician are trying to reach may be substantially different from the standard range. For example, the standard range for glycated hemoglobin (HbA_1c_) is generally 4.5%-5.7%, but a common recommendation for patients with type 2 diabetes is to aim to have their HbA_1c_ values below 7%. Furthermore, there is evidence that aggressively managing type 2 diabetes (HbA_1c_ goal: <6.0%) in older individuals results in increased mortality compared with standard therapy (HbA_1c_ goal: 7.0%-7.9%) [6-9], suggesting that at least some patients (eg, those experiencing frequent hypoglycemia) may need to be told that their HbA_1c_ values are lower than advisable. Even in situations where the patient may not be physically harmed by trying to reach the standard range, if the standard range is not realistically achievable, patients may feel justifiably frustrated and discouraged. This could lead to decreased motivation for self-management or the pursuit of alternative therapies in an effort to achieve the unachievable. In an attempt to avoid these potential harms, health care providers frequently discuss goal ranges with their patients that may be more realistic for a person with their condition. Goal ranges may also change with new evidence or changing life circumstances; thus, it may be important to have new ways to communicate these goal ranges via the patient portal.

In addition to the use of clinically appropriate goal ranges, use of visual displays could help increase patient sensitivity to variations among out-of-range results. In a previous study by our research group, we tested the impact of presenting laboratory test results via 3 number line formats versus a standard table format on participants’ sense of urgency and desire to contact their health provider [10]. Compared with participants in the table condition, participants in the 3 number line displays had reduced perceived urgency and desire to contact their health provider for test result values outside of, but near, the standard range. Furthermore, the use of visual displays did not affect participants’ perceived urgency and desire to contact their health provider about more extreme test values.

These issues raise the question of how can test results be communicated to patients in ways that help them better understand how their result compares to the target range most relevant to their self-management and treatment decision making. To the best of our knowledge, there has been no research examining whether and how individual- or disease-specific goal range information should be incorporated into the returned laboratory test results for patients such as these. Inclusion or exclusion of different combinations of these reference standards might improve patients’ comprehension of the test value and reduce unnecessary negative reactions, such as discouragement or urgency to contact their health care provider when urgency is unnecessary.

We conducted a Web-based experiment in which respondents imagined receiving HbA_1c_ test results through an EHR patient portal as part of the ongoing management of their type 2 diabetes. This study was designed to answer four key questions:

- Does the inclusion of goal range information improve comprehension of the test results?
- Does the inclusion of goal range information reduce unnecessary negative reactions to test results that are outside of the standard range, but near their goal range?
- Is it better to include the goal range information in addition to, or in place of, the standard range?
- Does the display format (eg, table vs visual number line) change the impact (if any) of including goal range information in the test result display?

Utilizing the principle “less is more,” which has been shown to apply in health communication [11-14], we hypothesized that the goal information would have the largest improvements in comprehension and reducing unnecessary negative reactions when the goal range was the only reference category (ie, conditions where the standard range and any other risk categories are absent). We also hypothesized that the impact of goal information would be most effective for values nearer to, but still outside of, the standard range because higher test values would be comparatively easier to interpret without additional information.

## Methods

### Setting

Data were collected through Qualtrics survey software (Qualtrics; Provo, UT) from a nationwide sample of US adults through Survey Sampling International (SSI). Participants were recruited over a 2-month period from August to October 2015.

### Sample

Participant eligibility was determined through SSI using a probability-weighted random process based on sample requirements. We established quotas on respondent age (33% aged 21-39 years, 33% aged 40-49 years, and 33% aged ≥60 years), gender (50% females), and race or ethnicity (14% African American, 14% Hispanic, and 4% Asian American people) to approximate the distribution of these characteristics in the US population. However, we oversampled individuals with diabetes to ensure that we did not have an overly healthy sample and to evaluate whether experience managing diabetes moderated the impact of the goal presentation format. SSI participants were routed to the survey via the sampling algorithm until all quotas were achieved.

### Design and Procedure

Participants were asked to imagine that they had recently visited their doctor’s office to discuss the management of their type 2 diabetes, during which their doctor had highlighted that people with type 2 diabetes should try to have HbA_1c_ values within a target or goal range of 6.5%-7.5%. Participants were then asked to imagine that in the intervening 3 months, they did their best to follow their doctor’s recommendations (eg, exercising regularly and eating healthy). Then, 3 months prior to their next appointment, the patients underwent some blood tests and viewed the results of these tests a day later via a Web-based EHR portal.

**Figure 1:**
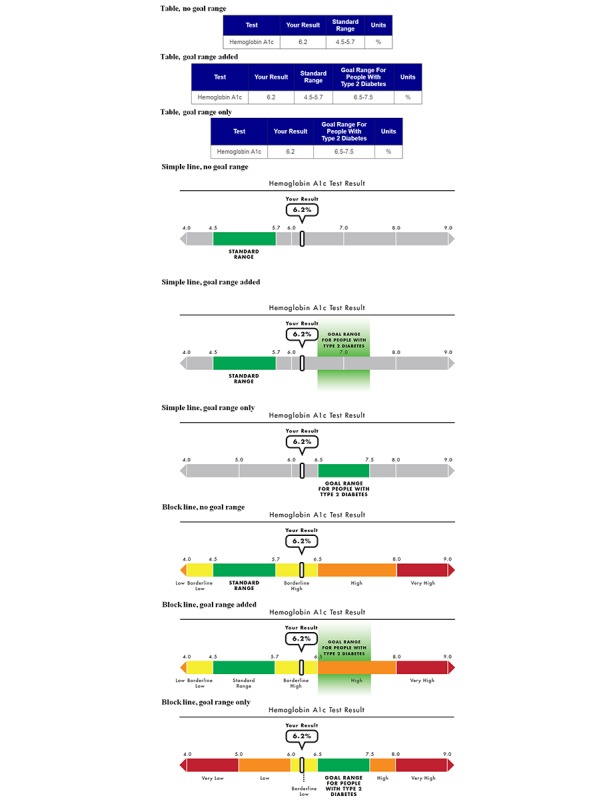
Goal presentation and display formats for 6.2% glycated hemoglobin (hemoglobin A_1c_) test value; labels indicate the display format and goal presentation.

We tested 3 between-subjects factors (varied independently) to examine the impact of including goal range information across different presentation formats on patient reactions to their test results. Figure 1 shows examples of different levels of each factor. The first factor was goal presentation. Approximately one-third of participants were randomly presented with a test result display with no goal range (standard range only condition), although the goal range information was described in the scenario text. The remaining participants received visual displays with the goal range included, either in addition to the standard range (goal range added condition) or with the goal range presented instead of the standard range (goal range only condition). The goal range was chosen in consultation with clinicians on our research team who care for patients with type 2 diabetes to represent a realistic and clinically appropriate target range for most individuals with type 2 diabetes.

The second factor was HbA_1c_ test value. Participants were randomly presented with an HbA_1c_ test value of 6.2%, which fell between the standard range and the goal range, or a value of 8.2%, which was higher than both the standard and goal ranges. The third factor was the display format**.** The HbA_1c_ test result was randomly presented via 1 of the 3 formats. The table format presented information via text in a table, the format typically used in EHRs. The simple line format was a gray number line, except for a green range labeled “standard range.” The block line format was a number line divided into differently colored diagnostic ranges. The cutoffs for the diagnostic categories were determined in consultation with clinician team members and differed for the “goal range only” condition compared with the “standard range only” and “goal range added” conditions to reflect the differences in hypoglycemia risk for individuals with type 2 diabetes compared with the general population. We reviewed our designs in color vision difference simulators to ensure that the different colors were distinguishable for people with color vision differences. For the “block line” plus “goal range only” combinations, the “Borderline Low” label was represented differently (dropped down, with dotted line connection) as a result of the label being greater in length than the range on the number line.

### Measures

#### Comprehension

We included 2 measures to assess how well participants understood their test result in relation to their goal range. For the relative location measure, we asked, “Where was your test result compared to your goal range?” with “higher than the goal range,” “within the goal range,” “below the goal range,” and “I don’t know” as response options. For the future location measure, we asked, “At your next test, what do you think your next test result should be, as compared to this test result?” using a 9-point Likert scale response option with “A lot lower” and “A lot higher” as the anchor labels and “About the same” as the midpoint label. “I don’t know” was also included as an additional response option.

#### Reactions to Test Result

We included 2 measures to assess participant reactions to their test result: one measuring how discouraged they would be by their test result and one assessing whether and when they would contact their doctor about their test result. For the discouraged measure, we asked, “How discouraged or encouraged do you feel about this test result?” using a 6-point Likert scale response option with “Very discouraged” and “Very encouraged” as the anchor labels with an additional “I don’t know” response option. For the urgency measure, we asked, “How soon do you need to speak to your doctor regarding these results?” with “Immediately,” “Within a few weeks,” “At your next appointment in 3 months,” and “I don’t need to speak to my doctor about these results” as response options.

#### Demographics

We asked participants about their age, gender, race and ethnicity, education, and whether they have diabetes, and if so, what type.

### Data Management

All data were collected anonymously so that the researchers had no way to learn the identity of the participants. A unique identification number provided by SSI was contained in the redirected URL, which identified participants and prevented them from completing the study multiple times. This study was deemed exempt by the University of Michigan Health Sciences and Behavioral Sciences Institutional Review Board.

### Data Analysis

#### Recoding of Measures

Responses to the relative location measure were recoded as “1” to indicate a correct response if participants responded “below the goal range” in the 6.2% HbA_1c_ test result condition or “higher than the goal range” in the 8.2% HbA_1c_ test result condition. All other responses were recoded as “0” to indicate a failure to know where their test value was in relation to the goal range. To assess whether participants had the gist of where their next test value should be, future location responses were recoded as “1” if they were above the midpoint of the scale in the 6.2% HbA_1c_ test result condition and below the midpoint in the 8.2% condition. All other responses were recoded as “0.” The results are substantially the same, if not stronger (ie, larger effect sizes), if “about the same” is coded as “1”. The one exception is that having diabetes is associated with an increased comprehension of the future location for the goal presentation and display format logistic regression analysis. Responses to the discouraged and urgency measures were reverse coded, such that higher scores indicated greater discouragement and urgency, respectively. We recoded gender (0=male, 1=female), race (0=white, 1=nonwhite), and diabetes status (0=no diabetes, 1=diabetes).

#### Effects of Goal Presentation

We report percentages for the relative and future location measures and descriptive measures for the discouraged and urgency measures across the different factors. We used chi-square analyses to test for differences in percentages and independent sample *t* tests and one-way analyses of variance (ANOVAs) with post hoc comparisons using Bonferroni corrections for multiple comparisons to compare means. We also report the logistic regression results for the relative and future location measures and ordered logistic regression results for the discouraged and urgency measures, with age, gender, race, education, and diabetes as covariates to test whether including relevant covariates substantially changed the results from the chi-square test, *t* test, and ANOVA. All analyses were performed using Stata 14, and all tests of significance were 2-sided and used alpha=.05.

## Results

### Sample Description

Of all the participants who initiated the study, 83.09% (6781/8161) completed it. In addition, 14 responses were dropped due to a reported age <18 years old, and 1 response was dropped due to a reported age of 586. Table 1 presents sample demographic characteristics among the remaining 6766 participants.

### Impact of Goal Presentation on Interpretation of Tables

**Figure 2:**
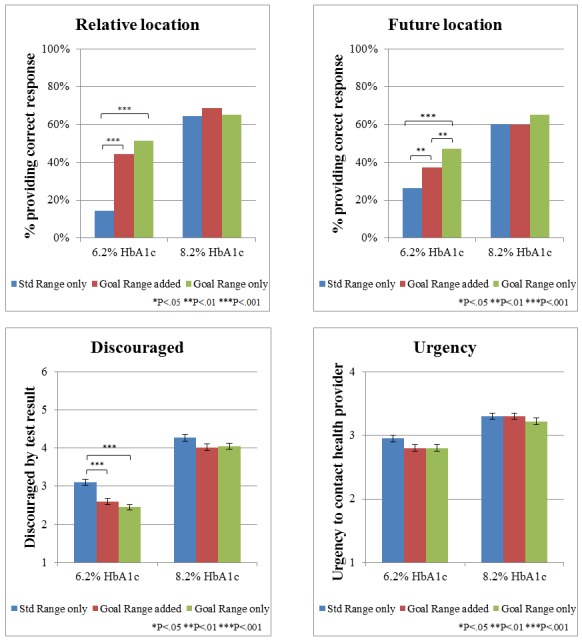
Effect of providing goal range information in table format, by goal presentation type and glycated hemoglobin (HbA_1c_) test result; asterisks indicate statistically significant differences between the 2 bars. Std range: standard range.

In univariate analyses of participants receiving HbA_1c_=8.2% in table form, neither goal presentation nor display factors significantly affected any of the outcomes (all *P* values>.07, see Figure 2).

Among participants who received tabular displays of HbA_1c_=6.2% results (which fell between the standard and goal ranges), however, goal presentation format had a significant impact on comprehension. As shown in Figure 2, receiving explicit goal information (in either form) significantly increased the percentage of participants recognizing that their HbA_1c_=6.2% value was below the goal range (*χ*^2^_2_=126.9, *P<*.001) and stating that their next result should be higher (*χ*^2^_2_=36.0, *P<*.001). Furthermore, the effect was larger among participants who viewed a table with the goal range only versus when the goal range was added to the standard range (relative location, goal range only: 51.28%, 201/1130, vs goal range added: 44.16%, 155/1130; *χ*^2^_1_=3.8, *P*=.05; future location, goal range only: 46.97%, 186/1137, vs goal range added: 37.39%, 132/1137; *χ*^2^_1_=7.0, *P*=.01). Similarly, providing goal information (either format) in table displays reduced discouragement (*F*_2,1071_=19.38, *P*<.001) and urgency (*F*_2,1131_=3.09, *P*=.046) compared with no goal displays, although there was no significant difference between the goal range added versus goal only conditions.

The logistic regression analyses of participants receiving test results in table format (Table 2) confirmed significant main effects for HbA_1c_ test value and goal presentation for all 4 outcome measures (all *P* values<.001) with the exception of goal presentation for urgency, which became nonsignificant when controlling for the covariates (*χ*^2^_2_=5.5, *P=*.06). Consistent with the pattern seen in Figure 2, there were significant interactions between HbA_1c_ test value and goal presentation for relative location (*χ*^2^_2_=62.8, *P<*.001), future location (*χ*^2^_2_=11.4, *P=*.003), and discouragement (*χ*^2^_2_=7.2, *P=*.03), but not for urgency (*χ*^2^_2_=1.2, *P*=.54). In addition, individuals with diabetes had not only a lower likelihood of identifying the relative location but also lower discouragement relative to individuals without diabetes. Being older and female were associated with an increased likelihood of identifying the relative location, but decreased urgency. Additionally, being older was associated with increased discouragement while identifying as female was associated with an increased likelihood of correctly identifying where their next test result should be. Identifying as a person of color (nonwhite) was associated with a decreased likelihood of identifying the relative location. Higher education was associated with not only an improved comprehension of the relative location but also increased urgency.

**Table 1:** Sample characteristics (N=6766).

Characteristic		Value^a^
Age, mean (SD)		49.1 (15.8)
**Gender, n (%)**		
	Male	3299 (48.88)
	Female	3435 (50.90)
	Transgender or other	15 (0.22)
**Ethnicity, n (%)**		
	Hispanic (any race)	892 (13.26)
**Race^b^, n (%)**		
	White	5294 (78.24)
	African American	1002 (14.81)
	All other	654 (9.67)
**Education, n (%)**		
	<High school	135 (2.00)
	High school only	1065 (15.78)
	Some college or trade	2458 (36.41)
	Bachelor’s degree	2005 (29.70)
	>Bachelor’s degree	1087 (16.10)
**Diabetes status, n (%)**		
	No diabetes	3620 (53.79)
	Type 1 diabetes	497 (7.38)
	Type 2 diabetes	2613 (38.83)
**Goal presentation, n (%)**		
	Standard range only	2253 (33.30)
	Goal range added	2219 (32.80)
	Goal range only	2294 (33.90)
**Glycated hemoglobin test result value, n (%)**		
	6.2%	3390 (50.10)
	8.2%	3376 (49.90)
**Display format, n (%)**		
	Table	2251 (33.27)
	Simple line	2224 (32.87)
	Block line	2291 (33.86)

^a^Results reported only for those respondents who completed each question or measure.

^b^Respondents could mark more than one race.

**Table 2:** Logistic regression and ordered logistic regression results showing predictors of outcome measures, table condition only.

Predictors		Relative location		Future location		Discouraged		Urgency		
		OR^a^ (95% CI)	*P* value	OR (95% CI)	*P* value	OR (95% CI)	*P* value		OR (95% CI)	*P* value
**Goal presentation**										
	Standard range only	Reference		Reference		Reference		Reference		
	Goal range added	4.98 (3.46-7.17)	<.001	1.69 (1.23-2.32)	<.001	0.51 (0.39-0.67)	<.001		0.77 (0.59-1.00)	.049
	Goal range only	6.83 (4.77-9.76)	<.001	2.52 (1.86-3.42)	<.001	0.47 (0.35-0.59)	<.001		0.74 (0.57-0.95)	.02
**Glycated hemoglobin (HbA_1c_) test result value**										
	6.2%	Reference		Reference		Reference		Reference		
	8.2%	12.07 (8.30-17.54)	<.001	4.30 (3.13-5.91)	<.001	4.13 (3.12-4.46)	<.001		2.16 (1.63-2.86)	<.001
**Goal × HbA_1c_**										
	Goal range added × 8.2%	0.25 (0.15-0.40)	<.001	0.57 (0.37-0.87)	.01	1.40 (0.95-2.05)	.09		1.24 (0.84-1.84)	.28
	Goal range only × 8.2%	0.15 (0.09-0.24)	<.001	0.49 (0.32-0.75)	<.001	1.67 (1.15-2.44)	.01		1.16 (0.79-1.71)	.46
**Demographics**										
	Diabetes^b^	0.64 (0.52-0.77)	<.001	1.03 (0.87-1.23)	.72	0.52 (0.44-0.61)	<.001		0.98 (0.84-1.15)	.83
	Age^c^	1.02 (1.01-1.03)	<.001	1.00 (0.99-1.00)	.54	1.01 (1.01-1.02)	<.001		0.98 (0.97-0.99)	<.001
	Gender^d^ (female)	1.74 (1.44-2.11)	<.001	1.03 (1.03-1.47)	.02	1.16 (0.99-1.35)	.06		0.84 (0.72-0.99)	.04
	Race^e^	0.70 (0.56-0.87)	.002	1.00 (0.81-1.24)	.99	1.01 (0.84-1.22)	.92		1.08 (0.90-1.32)	.39
	Education^c^	1.11 (1.05-1.17)	<.001	1.03 (0.98-1.08)	.24	0.99 (0.94-1.03)	.57		1.08 (1.04-1.13)	<.001
Constant		0.03 (0.02-0.05)	<.001	0.28 (0.16-0.47)	<.001	N/A^f^	N/A	N/A		N/A

^a^OR: odds ratio.

^b^Diabetes (0=no, 1=yes).

^c^Age and education treated as continuous variables.

^d^Gender (0=male, 1=female).

^e^Race (0=white, 1=nonwhite).

^f^N/A: not applicable.

### Impact of Display Format on Goal Presentation: Glycated Hemoglobin 6.2% Condition Only

**Figure 3:**
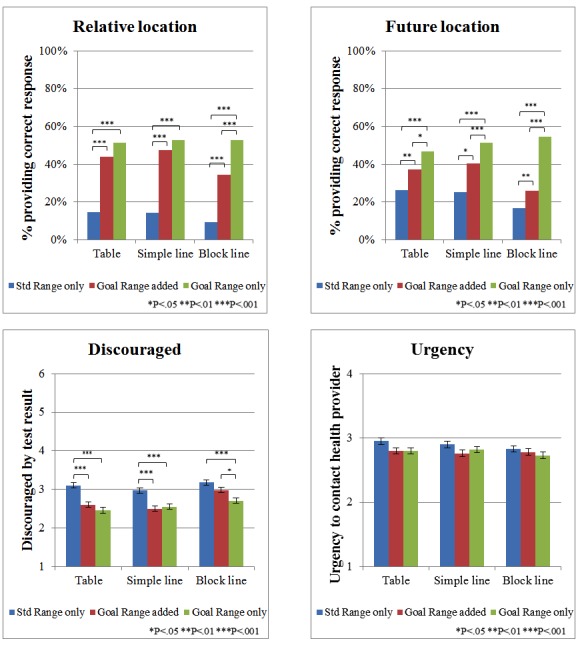
Effects of presenting goal information to patients viewing glycated hemoglobin 6.2% test result: goal presentation and display format; asterisks indicate statistically significant differences between the 2 bars. Std Range: standard range.

Given that providing goal information to participants receiving test results via tables only influenced outcomes among those viewing HbA_1c_=6.2% results, we focused only on these conditions when comparing optimal formats (ie, table vs simple line vs blocks line) for presenting goal information. As shown in Figure 3, the overall pattern of goal presentation on the understanding and interpretation of HbA_1c_=6.2% results in the simple line and block line conditions mirrored the pattern discussed above for the table format: providing goal information (in any format) increased the percentage of participants recognizing that their HbA_1c_ value was below the goal range, wanting their next result to be higher and experiencing less discouragement; however, there were minor differences with the block line design. Compared with the table and simple line designs where the goal range was added, participants in the block line condition exhibited less comprehension of their goal location (*χ*^2^_2_=13.9, *P<*.001) and where their next test result should be (*χ*^2^_2_=19.4, *P<*.001) as well as exhibited a greater discouragement *F*_2,1026_=11.42, *P*<.001).

The logistic regression analyses (Table 3) revealed the main effects of goal presentation (all *P* values ≤.002), with participants in the goal range added and goal range only conditions having higher comprehension and less discouragement and urgency than participants in the no goal condition and display type (all *P* values≤.002 for relative location, future location, and discouragement measures). More interestingly, there were also significant interactions between goal presentation and display format for relative location (*χ*^2^_4_=11.6, *P*=.02) and future location (*χ*^2^_4_=22.2, *P<*.001), but not for discouragement (*χ*^2^_4_=8.0, *P*=.09) or urgency (*χ*^2^_4_=3.2, *P*=.53). As noted earlier, the block line design seemed to interfere with the efficacy of including goal information when the standard range was also present (Figure 3).

**Table 3:** Logistic regression results showing goal presentation, presentation format, and demographics as predictors of outcome measures, 6.2% glycated hemoglobin test value condition only.

Predictors		Relative location				Future location			Discouraged				Urgency		
		OR^a^ (95% CI)		*P* value		OR (95% CI)		*P* value	OR (95% CI)		*P* value		OR (95% CI)		*P* value
**Goal presentation**															
	Standard range only	Reference				Reference			Reference			Reference			
	Goal range added	4.85 (3.38-6.96)		<.001		1.73 (1.25-2.39)		<.001	0.45 (0.34-0.59)	<.001		0.74 (0.56-0.96)		.03	
	Goal range only	6.6 (4.65-9.44)		<.001		2.65 (1.94-3.63)		<.001	0.39 (0.30-0.51)	<.001		0.72 (0.55-0.93)		.01	
**Presentation format**															
	Table	Reference				Reference			Reference			Reference			
	Simple line	1.02 (0.68-1.54)		.92		1.01 (0.73-1.42)		.93	0.85 (0.65-1.11)	.23		0.92 (0.71-.20)		.55	
	Block line	0.61 (0.39-0.97)		.04		0.60 (0.41-0.86)		.01	1.17 (0.90-1.53)	.25		0.83 (0.64-1.08)		.16	
**Goal ×** **presentation**															
	Goal range added × simple line	1.17 (0.70-1.95)		.54		1.17 (0.73-1.85)		.51	1.16 (0.78-1.70)	.46		0.99 (0.68-1.46)		.97	
	Goal range added × block line	1.07 (0.62-1.86)		.82		1.02 (0.63-1.67)		.93	1.56 (1.07-2.28)	.02		1.22 (0.83-1.78)		.31	
	Goal range only × simple line	0.99 (0.60-1.64)		.97		1.19 (0.76-1.86)		.45	1.36 (0.94-1.97)	.11		1.20 (0.83-1.74)		.34	
	Goal range only × block line	1.74 (1.01-2.99)		.045		2.32 (1.45-3.71)		<.001	1.29 (0.89-1.86)	.18		1.09 (0.76-1.58)		.64	
**Demographics**															
	Diabetes^b^	0.64 (0.55-0.75)	<.001		0.77 (0.66-0.89)		<.001		0.31 (0.27-0.35)	<.001		0.74 (0.65-0.84)		<.001	
	Age^c^	1.01 (1.01-1.02)	<.001		0.97 (0.96-0.97)		<.001		1.00 (0.99-1.00)	.049		0.97 (0.97-0.98)		<.001	
	Female gender^d^	1.56 (1.33-1.82)	<.001		0.90 (0.77-1.05)		.19		1.04 (0.91-1.18)	.57		0.79 (0.69-0.89)		<.001	
	Race^e^	0.76 (0.63-0.92)	.01		1.02 (0.85-1.22)		.85		0.96 (0.82-1.12)	.62		1.20 (1.03-1.40)		.02	
	Education^c^	1.11 (1.06-1.16)	<.001		1.04 (1.00-1.09)		.07		0.98 (0.95-1.02)	.29		1.03 (0.99-1.06)		.14	
Constant		0.04 (0.03-0.07)	<.001		1.38 (0.87-2.19)		.17		N/A^f^		N/A	N/A			N/A

^a^OR: odds ratio.

^b^Diabetes (0=no, 1=yes).

^c^Age and education treated as continuous variables.

^d^Gender (0=male, 1=female)

^e^Race (0=white, 1=nonwhite)

^f^N/A: not applicable.

Demographic covariates remained significant predictors across the 4 outcome measures (see Table 3). People with diabetes were less likely to identify the relative location of their result, but they also had less discouragement and urgency compared with individuals without diabetes. Age produced inconsistent effects, with a high comprehension of relative location but low comprehension of future location along with lower discouragement and urgency. Identifying as female or white were both associated with an increased comprehension of relative location and with decreased urgency. Education was associated with increased comprehension of relative location.

### Interaction Analysis of the Impact of Diabetes Status: Glycated Hemoglobin 6.2% Condition Only

The regression results presented in Table 3 showed consistent main effects on comparing participants who have diabetes in real life versus those who did not. To explore whether diabetes status might interact with optimal display formats, we performed additional regression analyses including interaction terms based on diabetes status. These additional logistic regression and ordered logistic regression results revealed a significant interaction between diabetes status and goal presentation for comprehension of the relative and future locations (all *P* values<.001), but not for discouragement and urgency (all *P* values>.15; Multimedia Appendix 1). For comprehension of relative location, the overall relationship between the effect of goal presentation did not change based on whether someone had diabetes (no goal: 80/552, 14.49% vs goal range added: 171/476, 35.92% and goal range only: 219/504, 43.45%; *P*<.001) or not (no goal: 65/578, 11.25% vs goal range added: 278/595, 46.72% and goal range only: 385/646, 59.60%; *P*<.001); the effects were just more exaggerated for people without diabetes. For comprehension of the future location, there were significant differences between all 3 goal presentation conditions for participants without diabetes (no goal: 132/579, 22.80% vs goal range added: 243/604, 40.23% and goal range only: 364/649, 56.09%; *P*<.001). However, for people with diabetes, comprehension was significantly higher in the goal only condition (225/506, 44.47%) than in no goal (129/555, 23.24%; *P*<.001) and goal range added (127/477, 26.62%; *P*<.001) conditions, while there were no significant differences between the no goal and goal range added condition (*P*=.70).

## Discussion

Our data suggest that providing people with test results displays (tabular or visual) that include goal range information can alter their perceptions of their test results in important ways. While perceptions were generally unaffected by format when the result was above both the standard and goal ranges, perceptions were sensitive to format when the result was above the standard range but below the goal range. Comprehension of the below-target nature of this result was higher when goal information was explicitly included in participants’ test result tables or visual displays. Furthermore, inclusion of goal information in the display reduced perceived discouragement about the presented results.

Our data also show that removing the standard range and substituting it with a single goal reference range seems superior to simply adding goal range information along with the standard range values. Comprehension was highest and discouragement and urgency were lowest when the goal range information was presented in lieu of the standard range information. This suggests that it is difficult for people to put aside information about the standard range—which is normed based on the total, mostly healthy, population—even when more personalized goal information is easily available. As a result, the inclusion of these standard reference points (which are less relevant in this particular situation) may undermine patients’ ability to manage their chronic conditions and may expose them to harm when aggressively trying to achieve test results within the standard range [6-9].

Fundamental principles of both visual design and information evaluability suggest that the dominance of the goal only substitution condition is due to the fact that the inclusion of more than 1 reference range produces confusion about which comparator is most relevant to understanding where the patient’s test value should be [15,16]. This argument is bolstered by the fact that among the conditions where goal information was presented in addition to the standard range, comprehension was lowest and discouragement was highest when participants received block design visuals. This design already includes multiple color-coded sections and categorical labels indicating levels of risk, and adding yet another reference range for patients to interpret at the same time was clearly too much for many to handle.

One limitation of our study is the use of a hypothetical scenario. While participants did not receive actual test results, approximately half of our sample had the medical condition described in the scenario (diabetes) and would likely have experience receiving HbA_1c_ test results. While we found the same pattern of results for participants with and without diabetes, participants with diabetes who received HbA_1c_=6.2% results were less likely to report that their values were too low, but these participants also exhibited decreased discouragement and urgency. One possible explanation for this finding is that their experience with repeatedly being told that their HbA_1c_ goal should be below 7.0% has led them to adopt the standard range as the norm that they should be striving to attain, even when an alternative goal range has been provided. Another possibility is that participants with diabetes were relying on their real-life goal ranges, which may have been different from the one provided in the scenario, or that they recognize that not all persons with type 2 diabetes will experience adverse outcomes with an HbA_1c_ of 6.2%. This explanation may account for the overall smaller percentage of participants with diabetes who were discouraged about their test result or felt a need to contact their health care provider immediately.

As more and more patients receive their test results via Web-based patient portals, it is becoming increasingly important that patients should be able to find their results meaningful and that we do not cause unnecessary distress or discouragement to patients. Current approaches to presenting laboratory test results to patients appear to be particularly problematic for many patients, such as those with chronic conditions, who may have personal target goals that differ from those relevant to healthy adults. For these patients, the standard range commonly shown is not necessarily where we want patient results to be. Providing goal range information in place of the standard range may be one step toward reducing these problems with EHR systems; however, challenging discussions would need to occur regarding the pros and cons of who should determine the goal range information (ie, health systems, EHR or portal vendors, expert panels, individual physicians, and/or patients) or what the goal ranges should represent (eg, broader goals for people with a chronic condition vs individualized goals). More research is needed to determine additional features that may further improve the interpretability of laboratory test results.

## Appendix

Multimedia Appendix 1 Logistic regression and ordered logistic regression results showing goal presentation, diabetes status, and demographics as predictors of outcome measures, 6.2% A_1c_ test value condition only.

## Abbreviations

ANOVA: analysis of variance
EHR: electronic health record
HbA _1c_: glycated hemoglobin
SSI: Survey Sampling International
