# Supplementary material for: Improving the Understanding of Test Results by Substituting (Not Adding) Goal Ranges: Web-Based Between-Subjects Experiment
Source: J Med Internet Res. 2018 Oct 19;20(10):e11027. doi: 10.2196/11027 (PMC6231727; doi:10.2196/11027)
Supplement: Multimedia Appendix 1 [file jmir_v20i10e11027_app1.pdf]

Multimedia Appendix

Logistic regression and ordered logistic regression results showing goal presentation, diabetes status, and demographics as predictors of outcome measures, 6.2% A1c test value condition only.

	Relative location<br>OR (95% CI)<br>P value	Future location<br>OR (95% CI)<br>P value	Discouraged<br>OR (95% CI)<br>P value	Urgency<br>OR (95% CI)<br>P value
**Goal presentation**				
Standard Range only	reference	reference	reference	ref reference
Goal Range added	7.26 (5.32, 9.92)<br>*P*<.001	2.48 (1.91, 3.23)<br>*P*<.001	0.57 (0.46, 0.71)<br>*P*<.001	0.71 (0.57, 0.89)<br>*P*=.01
Goal Range only	12.37 (9.08, 16.87)<br>*P*<.001	4.84 (3.73, 6.27)<br>*P*<.001	0.50 (0.41, 0.61)<br>*P*<.001	0.68 (0.55, 0.84)<br>*P*<.001
**Diabetes Status**				
No diabetes	reference	reference	reference	reference
Diabetes	1.31 (0.91, 1.87)<br>*P*=.14	1.25 (0.94, 1.66)<br>*P*=.13	0.33 (0.26, 0.41)<br>*P*<.001	0.63 (0.51, 0.78)<br>*P*<.001
**Goal x *P*resentation**				
Goal Range added x Diabetes	0.47 (0.30, 0.73)<br>*P*<.001	0.48 (0.32, 0.71)<br>*P*<.001	0.98 (0.72, 1.33)<br>*P*=.89	1.21 (0.88, 1.65)<br>*P*=.24
Goal Range only x Diabetes	0.37 (0.24, 0.57)<br>*P*<.001	0.54 (0.37, 0.78)<br>*P*<.001	0.91 (0.67, 1.23)<br>*P*=.53	1.34 (0.99, 1.81)<br>*P*=.06
**Demographics**				
Age	1.01 (1.01, 1.02)<br>*P*<.001	0.97 (0.96, 0.97)<br>*P*<.001	1.00 (0.99, 1.00)<br>*P*=.10	0.97 (0.97, 0.98)<br>*P*<.001
Female Gender	1.57 (1.34, 1.84)<br>*P*<.001	0.91 (0.78, 1.06)<br>*P*=.23	1.03 (0.91, 1.17)<br>*P*=.65	0.78 (0.69, 0.89)<br>*P*<.001
Race	0.77 (0.63, 0.93)<br>*P*=.01	1.03 (0.86, 1.24)<br>*P*=.72	0.95 (0.82, 1.11)<br>*P*=.54	1.20 (1.03, 1.40)<br>*P*=.02
Education	1.11 (1.07, 1.17)<br>*P*<.001	1.04 (1.00, 1.09)<br>*P*=.06	0.98 (0.95, 1.02)<br>*P*=.33	1.03 (0.99, 1.06)<br>*P*=.14
**Constant**	0.03 (0.02, 0.04)<br>*P*<.001	0.97 (0.63, 1.49)<br>*P*=.88		

Note: OR, odds ratio; CI, confidence interval. Diabetes (0=no, 1=yes); gender (0=male, 1=female); race (0=White, 1=non-White); age and education treated as continuous variables.
